# A viral RNA-dependent RNA polymerase inhibitor VV116 broadly inhibits human coronaviruses and has synergistic potency with 3CLpro inhibitor nirmatrelvir

**DOI:** 10.1038/s41392-023-01587-1

**Published:** 2023-09-22

**Authors:** Yumin Zhang, Yuan Sun, Yuanchao Xie, Weijuan Shang, Zhen Wang, Hualiang Jiang, Jingshan Shen, Gengfu Xiao, Leike Zhang

**Affiliations:** 1 State Key Laboratory of Virology, Wuhan Institute of Virology, Center for Biosafety Mega-Science, Chinese Academy of Sciences, 430071 Wuhan, China; 2 University of Chinese Academy of Sciences, 100049 Beijing, China; 3 Lingang Laboratory, 200031 Shanghai, China; 4 Shanghai Institute of Materia Medica, Chinese Academy of Sciences, 201203 Shanghai, China; 5 Hubei Jiangxia Laboratory, 430200 Wuhan, China

**Keywords:** Drug screening, Drug safety

## Abstract

During the ongoing pandemic, providing treatment consisting of effective, low-cost oral antiviral drugs at an early stage of SARS-CoV-2 infection has been a priority for controlling COVID-19. Although Paxlovid and molnupiravir have received emergency approval from the FDA, some side effect concerns have emerged, and the possible oral agents are still limited, resulting in optimized drug development becoming an urgent requirement. An oral remdesivir derivative, VV116, has been reported to have promising antiviral effects against SARS-CoV-2 and positive therapeutic outcomes in clinical trials. However, whether VV116 has broad-spectrum anti-coronavirus activity and potential synergy with other drugs is not clear. Here, we uncovered the broad-spectrum antiviral potency of VV116 against SARS-CoV-2 variants of concern (VOCs), HCoV-OC43, and HCoV-229E in various cell lines. In vitro drug combination screening targeted RdRp and proteinase, highlighting the synergistic effect of VV116 and nirmatrelvir on HCoV-OC43 and SARS-CoV-2. When co-administrated with ritonavir, the combination of VV116 and nirmatrelvir showed significantly enhanced antiviral potency with noninteracting pharmacokinetic properties in mice. Our findings will facilitate clinical treatment with VV116 or VV116+nirmatrelvir combination to fight coronavirus infection.

## Introduction

Coronavirus (CoV) is a huge threat to public health worldwide, as illustrated by past SARS-CoV and MERS-CoV outbreaks and the ongoing COVID-19 pandemic. In addition to the three highly pathogenic human CoVs (HCoVs) with high case fatality rates, four additional HCoVs, HCoV-OC43 (OC43), HCoV-229E (229E), HCoV-NL63 (NL63), and HCoV-HKU1 (HKU1), usually seasonally cause human upper respiratory tract infection. Despite these low virulence HCoVs leading to mild and self-limiting disease, they have a high probability of causing severe and even fatal diseases in children, the elderly, and immunocompromised patients. Some studies have demonstrated that the mutation rates of CoVs are moderate to high compared to those of other single-stranded RNA viruses,^1^ which indicates that CoVs are rapidly genetically recombining and evolving. Importantly, the SARS- and MERS-like CoVs circulating in bat populations can efficiently infect primary human airway cells, further demonstrating the potential for CoVs to exhibit cross-species transmission in the future.^2,3^

There has been no significant drug development to treat diseases caused by human CoVs since the first clinical CoV infection was discovered in the 1960s. RNA-dependent RNA polymerase (RdRp) is one of the most promising targets due to its pivotal role in viral RNA synthesis and the high sequence and structural conservation among CoVs. Therefore, nucleos(t)ide analogs might be the most valuable and broad-spectrum viral RdRp inhibitors due to the mechanisms by which RdRp identifies dNTPs and elongates nascent RNA chains. Remdesivir (RDV) is the first nucleoside analog drug approved by the FDA for COVID-19 therapy through intravenous administration, having positive clinical feedback for reduced recovery time but a nonsignificant trend toward lowering mortality.^4–6^ Molnupiravir (EIDD-2801) is an oral prodrug of β-d-N4-hydroxycytidine (NHC) that can inhibit SARS-CoV-2 replication via nucleoside analog incorporation into viral RNA, inducing lethal accumulation of mutations in the viral genome. Both remdesivir and molnupiravir showed broad-spectrum anti-coronavirus activities depending on the cell-based assay.^7,8^ However, the broad-spectrum activities of nucleos(t)ide analogs need to be further preclinically investigated further, especially in animal models, for their potential as pan-coronavirus drugs, and more promising RdRp inhibitors with oral availability should be developed.

**Fig. 1:**
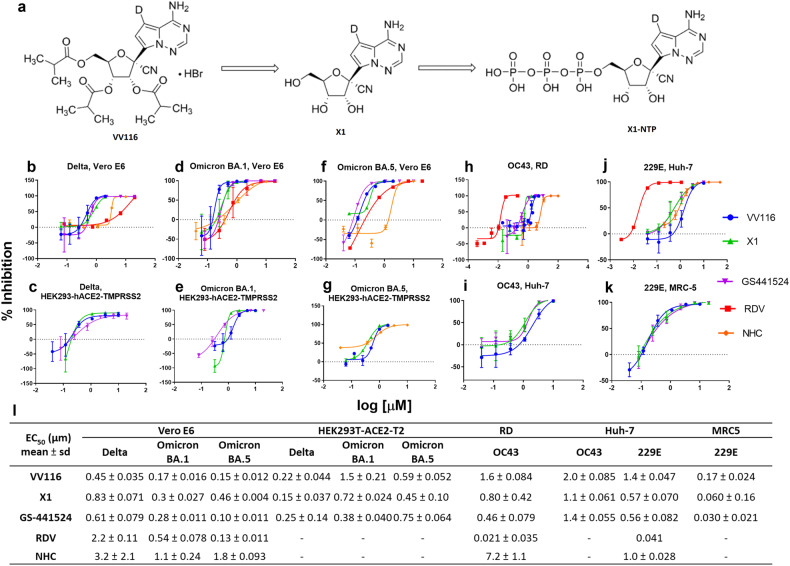
VV116 and its parent nucleoside X1 broadly inhibited human coronavirus. **a** The chemical structures of VV116 and X1. VV116 is efficiently metabolized to X1 after oral uptake, and then X1 is metabolized to active triphosphate X1-NTP in the cells. **b–k** The activity of VV116 in inhibiting SARS-CoV-2 variants (Delta, Omicron BA.1, and Omicron BA.5), HCoV-OC43, and HCoV-229E. The curves were fitted with a nonlinear regression model. VV116, X1, GS-441524, remdesivir (RDV), and NHC were compared head-to-head in Vero E6, RD, and Huh-7 cells (**b**, **d**, **f**, **h**, **j**), and then the antiviral activity of VV116, X1, and GS-441524 against the SARS-CoV-2 variants (Delta, Omicron BA.1, and Omicron BA.5), HCoV-OC43, and HcoV-229E were validated and compared in HEK293T-hACE2-TMPRSS2, Huh-7, and MRC-5 cells, respectively. Error bars denote mean ± sd of 3–6 independent replicates (**c**, **e**, **g**, **I**, and **k**). **l** The half-maximum effective concentration (EC_50_) values of compounds against HCoVs. The EC_50_ values were calculated using nonlinear regression in Prism version 7.00 (GraphPad software). The bar indicates the standard deviation (SD) from 3–6 independent experiments

As a ProTide prodrug, remdesivir must be administered intravenously because of its extensive hepatic first-pass metabolism. Considering the route limit of remdesivir, we previously reported on oral remdesivir derivative VV116, a tri-isobutyrate ester prodrug of the C7-deuterated GS-441524 analog (Fig. 1a), which had antiviral potency against the original SARS-CoV-2 strain in Vero E6 cells and hACE2-transduced mice.^9^ During a phase III clinical trial (NCT05341609), oral VV116 treatment given to adults with mild-to-moderate COVID-19 caused by the B.1.1.529 variant was non-inferior to 3C-like protease (3CLpro) inhibitor Paxlovid (nirmatrelvir–ritonavir) regarding the time to sustained clinical recovery, with fewer safety concerns.^10^ Therefore, VV116 is a promising weapon to fight the COVID-19 pandemic, and its broad-spectrum activity against SARS-CoV-2 variants and other HCoVs urgently needs investigation.

Although the COVID-19-approved drugs remdesivir, molnupiravir, and Paxlovid have demonstrated positive therapeutic outcomes in clinical trials, there are several unexpected problems regarding low efficacy and potential toxicity under real-world treatment.^11,12^ Drug cocktail therapy that can significantly improve therapeutic efficacy and prevent the development of drug resistance has become the standard treatment course for chronic and rapidly evolving viruses such as HIV and HCV. Although the highly pathogenic coronaviruses SARS-CoV, MERS-CoV, and SARS-CoV-2 cause severe acute respiratory syndrome in humans, other HCoVs, such as OC43, 229E, and HK1, can lead to long-term and repeated infection in which drug-resistant viruses are easily produced. For COVID-19, it has been reported that a SARS-CoV-2 isolated in the clinic with low susceptibility to remdesivir has emerged, and 1–2% of people treated with Paxlovid still tested positive after finishing the treatment, according to Pfizer’s clinical trial.^13,14^ Learning from the precedent of therapies against HIV or HCV, promising therapeutic drug combination regimens is urgently needed against current evolving SARS-CoV-2 and HCoVs that might emerge in the future. In this study, we first assessed the broad-spectrum potency of VV116 against HCoVs, including SARS-CoV-2 Delta and omicron variants in cell lines, and then found that VV116 plus nirmatrelvir showed synergistic antiviral effects through a quantifying screening of polymerase and protease inhibitor combinations. Furthermore, we estimated the broad-spectrum activity of VV116 and its enhanced effect when combined with nirmatrelvir in mouse models. This study is expected to provide an overall insight into the anti-HCoV potential of the nucleotide analog VV116 in both single and combination regimens.

## Results

### Evaluation of anti-HCoV activity of VV116 in vitro

To investigate the antiviral spectrum and activity of VV116 in cells, we evaluated its inhibitory activity against SARS-CoV-2 variants (Delta, Omicron BA.1, and Omicron BA.5), HCoV-OC43, and HCoV-229E in cell lines. We also compared the efficacy of VV116 and its parent nucleoside X1 with remdesivir, GS-441524, and β-d-N4-hydroxycytidine (NHC) in parallel. Each CoV was separately propagated in two cell lines for the antiviral activity assessment. Our results suggested that VV116 and X1 exhibited broad-spectrum anti-HCoV activity with half-maximal effective concentrations (EC_50_) ranging from 0.17 to 2.0 µM and 0.060–1.1 µM, respectively (Fig. 1b–l). The EC_50_ values of VV116 against the SARS-CoV-2 Delta and Omicron BA.1 variant in Vero E6 cells were 0.45 and 0.17 µM, respectively, exhibiting higher potency than remdesivir with EC_50_ values of 2.2 and 0.54 µM, respectively, and VV116 and remdesivir showed comparable activity against Omicron BA.5 (EC_50_ = 0.15 vs. 0.13 µM) (Fig. 1b–g, l). In RD and Huh-7 cells, X1 showed potency against OC43 and 229E similar to that of GS-441524, although the potency of VV116 was lower than that of remdesivir (Fig. 1h, i, l). VV116, X1, remdesivir, and GS-441524 displayed higher anti-CoV potency than NHC in this study. The remarkable antiviral activity of VV116 and X1 against these CoVs can be reproduced in HEK293T cells stably expressing the human angiotensin-converting enzyme 2 (ACE2) gene and the transmembrane serine protease 2 (TMPRSS2) gene (HEK293T-hACE2-TMPRSS2), as well as in human diploid MRC-5 cells (Fig. 1f–l). Cytotoxicity testing revealed that VV116 and X1 in different cell lines had half-maximal cytotoxic concentrations (CC_50_) of 43.4–146 and >125–>500 µM, respectively. The corresponding selectivity indices (SI = CC_50_/EC_50_) for VV116 and X1 of 43–566 and 156–8000, respectively; the CC_50_ and SI values of remdesivir, GS-441524, and NHC were also determined in parallel in different cell lines (Supplementary Fig. 1 and Supplementary Table 1).

### Drug combination screening showed that VV116 plus nirmatrelvir had synergistic antiviral potency in cells

**Fig. 2:**
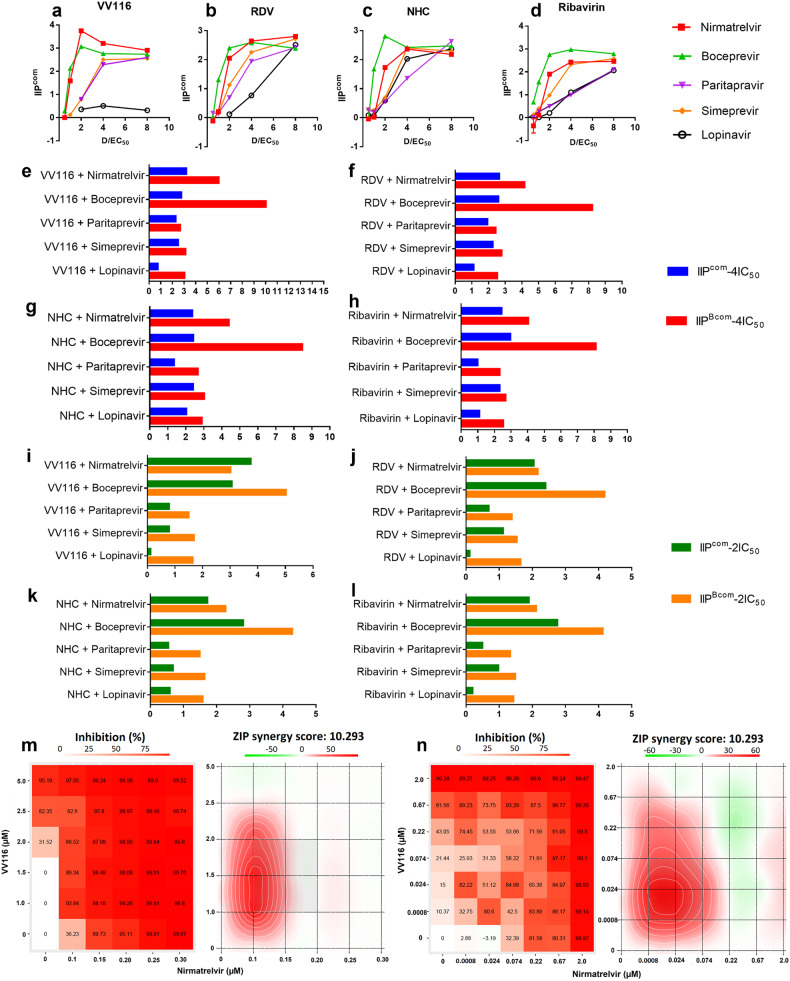
In vitro quantification of the antiviral activity of combinations of two drugs to screen high potency drug combinations against HCoV-OC43. **a–d** The instantaneous inhibitory potential (IIP) of combinations of nucleoside analogs and proteinase inhibitors. The concentrations of the drug combinations were normalized by IC_50_ values. D drug concentration. **e–h** Experimental IIP values (IIP^com^) and corresponding theoretical IIP values predicted by Bliss independence (IIP^Bcom^) of double drug combinations at 4 × IC_50_. **i–l** IIP^com^ and IIP^Bcom^ values of double drug combinations at 2 × IC_50_. The columns represent one of three independent experiments. **m** and **n** The landscapes for the interaction of VV116 and nirmatrelvir against HCoV-OC43 (**m**) and the SARS-CoV-2 Delta variant (**n**). The synergy δ-score was calculated using SynergyFinder with the zero-interaction potency (ZIP) model. Each point and column bar represent one of three independent experiments

Combinations of antiviral drugs targeting multiple distinct viral targets can potentially induce enhanced or synergistic antiviral effects, while also reducing the doses of individual drugs needed and resulting in fewer side effects.^15,16^ To develop oral drug combinations with higher potency than individual VV116 and other clinical drugs, we selected four nucleotide analogs (VV116, remdesivir, NHC, and ribavirin) and five viral protease inhibitors (nirmatrelvir, boceprevir, paritaprevir, simeprevir, and lopinavir) to produce combinations that would inhibit double viral targets with the expectation of achieving enhanced antiviral activity. Therefore, we screened 20 combinations of nucleotide analogs plus protease inhibitors for anti-OC43 activity in RD cells. The antiviral activity of individual drugs or drug combinations was quantified according to their instantaneous inhibitory potential (IIP), which combines three parameters: the drug concentration, the slope parameter of the dose–response curve, and the drug concentration that inhibited 50% of viral replication (IC_50_). A higher IIP reflects higher antiviral activity (see the section “Materials and methods”). We found that drug combinations displayed various IIP values against OC43 (Supplementary Fig. 2 and Supplementary Table 2). The drug combinations gained increased IIP values (IIP_com_) with increased drug concentrations, but IIP_com_ tended to plateau or even decrease when the drug concentrations continuously increased (Fig. 2a–d). Most double combinations did not reach the theoretical IIP values predicted by their Bliss independence (IIP_B_) at both 2× and 4×IC_50_ concentrations. However, the combinations of VV116 and nirmatrelvir exhibited higher antiviral activity than the synergistic activity predicted at a 2×IC_50_ concentration (IIP = 3.7 vs. IIP_B_ = 3.0) (Fig. 2e–l). To validate the enhanced anti-CoV activity of the VV116 plus nirmatrelvir combination, we performed antiviral combination assays on HCoV-OC43, the SARS-CoV-2 Delta variant, and the SARS-CoV-2 Omicron BA.5 variant by using the zero-interaction potency model (ZIP) for synergy assessment. The interaction landscapes showed that the VV116 and nirmatrelvir combination against HCoV-OC43 and the SARS-CoV-2 Delta and Omicron BA.5 variant had a synergistic interaction covering most of the concentration areas, with ZIP synergy scores from 4.76 to 10.382, respectively (Fig. 2m, n and Supplementary Fig. 3). Thus, the VV116 and nirmatrelvir combination enhanced the inhibitory activity against HCoV-OC43 and the SARS-CoV-2 Delta and Omicron BA.5 variant. It is not clear why nirmatrelvir synergized with VV116 instead of remdesivir against HCoV-OC43, the difference in the synergy performance might result from their distinct cytotoxicity in RD cells (the CC_50_ values of VV116 and remdesivir were 138 and 1.57 µM, respectively, Supplementary Fig. 2).

### Therapeutic efficacy of VV116 and VV116 plus nirmatrelvir combination against HCoV-OC43 in vivo

**Fig. 3:**
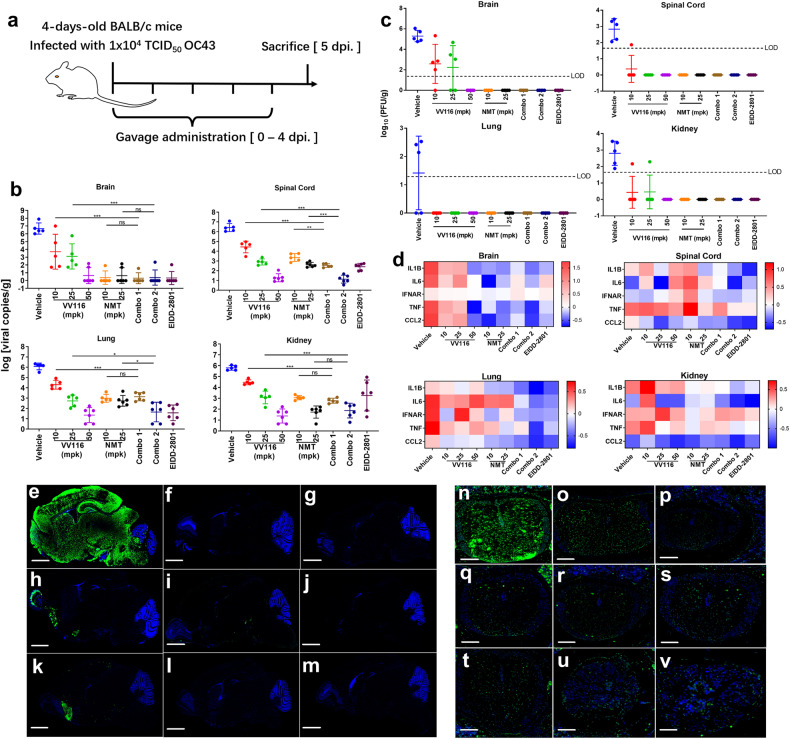
In vivo efficacy of VV116, nirmatrelvir, and the VV116 plus nirmatrelvir combination in 5-day-old suckling mice infected with HCoV-OC43. **a** Schematic of the experimental design for therapeutic treatment in suckling mice. Mice were intranasally challenged with 10^4^ TCID50 of HCoV-OC43. Mice were divided into 9 groups (*n* = 5 for each group): the vehicle group, the group receiving VV116 10 mpk, the group receiving VV116 25 mpk, the group receiving VV116 50 mpk, the group receiving nirmatrelvir 10 mpk with ritonavir 50 mpk, the group receiving nirmatrelvir 25 mpk with ritonavir 50 mpk, the group receiving drug combination of VV116 10 mpk and nirmatrelvir 10 mpk with ritonavir 50 mpk (Combo 1), the group receiving drug combination of VV116 25 mpk and nirmatrelvir 25 mpk with ritonavir 50 mpk (Combo 2), or the group receiving EIDD-2801 200 mpk. Vehicle or drug was administered at 2 h post-infection, and then quaque die (q.d.) from day 1 to day 4. Lung tissues were collected at 5 days post-infection (*n* = 5). **b** Determination of viral RNA copies targeting nucleoprotein genes in the brains, spinal cords, lungs, and kidneys collected on day 5 by real-time fluorescence quantitative PCR. **c** Determination of viral titers in the brains, spinal cords, lungs, and kidneys collected on day 5 by immunoplaque assay. **d** Cytokine gene expression was measured in the brain, spinal cord, lungs, and kidneys at day 5. The relative gene expression of IL-1β, IL-6, IFNAR, TNF-α, CCL2, CXCL10, and ISG15 was compared to that of unchallenged mice. **e–v** Immunofluorescence staining of brains and spinal cords to detect the SARS-CoV-2 antigen. **e**, **n** Vehicle, **f**, **o** EIDD2801-200 mpk, **g**, **p** VV116-50 mpk, **h**, **q** VV116-10 mpk, **i**, **r** nirmatrelvir-10 mpk + rito-50 mpk, **j**, **s** Combo 1, **k**, **t** VV116-25 mpk, **l**, **u** nirmatrelvir-25 mpk with ritonavir-50 mpk, and **m**, **v** Combo 2. The scale bars on the pictures of tissue slides indicate 1000 µm. The data on viral copies and viral titers were statistically analyzed with Student’s *t*-test. *P < 0.05; **P < 0.01; ***P < 0.001; and ns, not significant

To assess the antiviral activity of VV116 and the VV116 plus nirmatrelvir combination in vivo, we initially tested the therapeutic efficacy of VV116 against HCoV-OC43 in suckling Balb/c mice. HCoV-OC43 can cause lethal infection in suckling C57BL/6J and Balb/c mice by inducing acute encephalitis.^17,18^ Here, we orally treated 5-day-old suckling Balb/c mice that were intranasally challenged with HCoV-OC43 with VV116, nirmatrelvir (with 50 mpk ritonavir), and a combination of VV116 and nirmatrelvir at 2 h post-infection (hpi) (Fig. 3a). Molnupiravir (EIDD-2801) was employed as a positive control at a dose of 200 mpk. All the groups were treated with drugs, drug combinations, or the vehicle once a day. At 6 days post-infection (dpi), viral RNA copies in the vehicle-treated group reached 10^6^–10^8^ copies/g in the brain, spinal cord, lung, and kidney (Fig. 3b). Treating with VV116 (10 mpk, 25 mpk, and 50 mpk) resulted in a dose-dependent 2–4 log decrease in viral RNA load in the spinal cord, lung, and kidney (Fig. 3b). The groups treated with nirmatrelvir showed a similar effect to that of VV116. Except for VV116 and nirmatrelvir at 10 mpk, treating with VV116 at doses of 25 mpk and 50 mpk and nirmatrelvir at a dose of 25 mpk almost completely cleared the viral copies in the brain (Fig. 3b). Interestingly, the combination treatments consisting of VV116 at 10 mpk plus nirmatrelvir at 10 mpk (Combo 1) and VV116 at 25 mpk plus nirmatrelvir at 25 mpk (Combo 2) showed significantly higher antiviral potency than individual drugs in specific organs. Combo 2 treatment greatly reduced the viral copies by 4 logs in the lung and 5 logs in the spinal cord compared to the vehicle-treated group, while individual VV116 or nirmatrelvir reduced viral copies by 3 logs in the lung and spinal cord compared to the vehicle-treated group (Fig. 3b). Combo 1 treatment also showed enhanced potency in the spinal cord (Fig. 3b). Both Combo 1 and Combo 2 were not more effective at reducing the viral copies than individual nirmatrelvir treatments in the brain and kidney (Fig. 3b). Further testing revealed that the viral loads of VV116 at 50 mpk, nirmatrelvir at 10 and 20 mpk with ritonavir, and the two combinations were reduced to less than the limit of detection (LOD) in organs (Fig. 3c). Immunofluorescence staining of the nucleoprotein of HCoV-OC43 in the central nervous system (brain and spinal cord) confirmed that the VV116 and nirmatrelvir combination presented enhanced antiviral potency (Fig. 3e–v). A gene expression analysis of cytokines (IL-1β, IL-6, IFNAR, TNF-α, CCL2, CXCL10, and ISG15) revealed that inflammation produced by HCoV-OC43 infection was severe in the brain, lungs, spinal cord, and kidneys (Fig. 3d). VV116 (50 mpk) and nirmatrelvir (10 mpk, 25 mpk) dramatically reduced inflammation in the brain, but both the VV116 and nirmatrelvir treatments resulted in limited inflammation suppression, especially IL-6 suppression in the lungs. In contrast, Combo 1 and Combo 2 treatments resulted in strong inflammation reduction (Fig. 3d). Histopathological images from hematoxylin and eosin stains of lung tissues in response to HCoV-OC43 infection (vehicle group) showed prominent histiocytic perivascular infiltrates and alveolar septal thickening; by contrast, VV116 (50 mpk), nirmatrelvir (10 mpk, 25 mpk), Combo 1, and Combo 2 significantly improved the lung histopathology (Supplementary Fig. 4).

### Validating the antiviral activity of the drug combination against the SARS-CoV-2 Delta variant in the K18-hACE2 mouse model

**Fig. 4:**
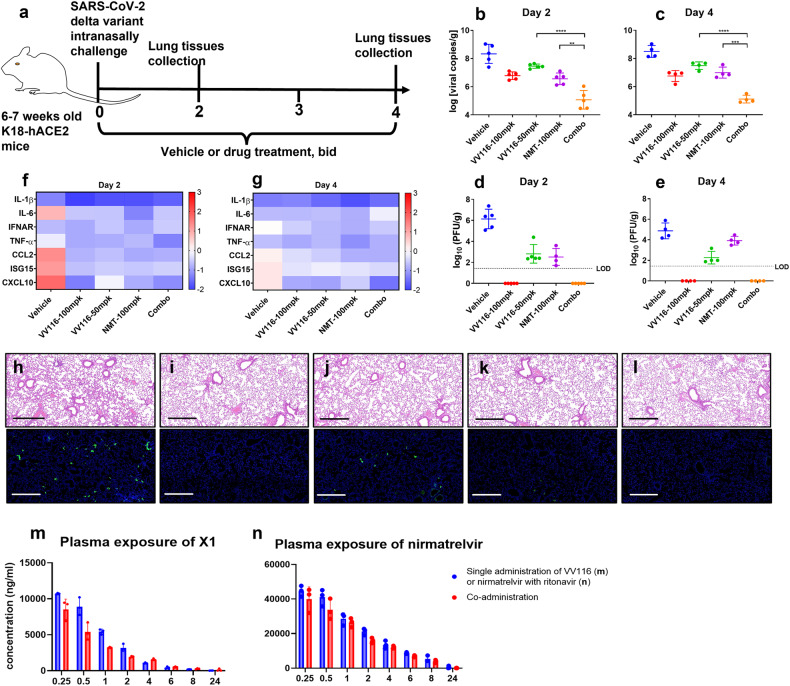
In vivo efficacy of VV116, nirmatrelvir, and the VV116 plus nirmatrelvir combination in K18-hACE2 mice infected with the SARS-CoV-2 Delta variant. **a** Schematic of the experimental design for therapeutic treatment in K18-hACE2 mice. The mice were intranasally challenged with 1000 pfu of the SARS-CoV-2 Delta variant. The mice were divided into five groups (*n* = 9 for each group): the vehicle group, the group receiving VV116 100 mpk, the group receiving VV116 50 mpk, the group receiving nirmatrelvir 100 mpk with ritonavir 50 mpk, and the group receiving the drug combination (Combo) of VV116 50 mpk and nirmatrelvir 100 mpk with ritonavir 50 mpk. The vehicle or drugs were orally administered at 2 h post-infection and were then administered bis in die (b.i.d.) at 8-h intervals from day 1 to day 4. Lung tissues were collected at 2 days post-infection (*n* = 5) and 4 days post-infection (*n* = 4). **b** and **c** Determination of viral RNA copies targeting the gene of receptor binding domain in the lungs collected at day 2 and day 4 by real-time fluorescence quantitative PCR. **d** and **e** Determination of viral titers in lungs collected at day 2 and day 4 by plaque assay. **f** and **g** Cytokine gene expression was measured in the lungs at days 2 and 4. The relative gene expression of IL-1β, IL-6, IFNAR, TNF-α, CCL2, CXCL10, and ISG15 was compared to that of unchallenged mice. **h–l** Histopathological analysis (hematoxylin-eosin) and immunofluorescence staining to detect the SARS-CoV-2 antigen in lung tissues of the vehicle (**h**), VV116 100 mpk (**i**), VV116 50 mpk (**j**), nirmatrelvir 100 mpk with ritonavir 50 mpk (**k**), and drug combination (Combo) VV116 50 mpk and nirmatrelvir 100 mpk with ritonavir 50 mpk (**l**) groups. Scale bars indicate 500 µm. **m** and **n** Plasma pharmacokinetic analysis of C57BL/6 J mice that received oral doses of VV116 (single administration), nirmatrelvir + ritonavir (single administration), and VV116 + nirmatrelvir + ritonavir (co-administration) at 50, 100 + 50, and 50 + 100 + 50 mg/kg, respectively. Data on the viral copies and viral titers were statistically analyzed with Student’s *t*-test. *P < 0.05; **P < 0.01; ***P < 0.001; ****P < 0.0001; and ns, not significant

In our previous study, VV116 showed high activity in hACE2-transduced mice following a challenge with the original SARS-CoV-2 strain.^9^ Compared to hACE2-transduced mice, transgenic mice expressing human angiotensin-converting enzyme 2 (hACE2) by the human cytokeratin 18 promoter (K18-hACE2) are highly susceptible to SARS-CoV-2 infection with a high viral load detected in multiple organs, which leads to lethal disease if no intervention is given.^19^ In this study, we further investigated VV116, particularly in combination with nirmatrelvir, against the SARS-CoV-2 Delta variant in a K18-hACE2 mouse model. Mice were intranasally infected with the SARS-CoV-2 Delta variant, and after 2 h, they were orally treated (BID) with vehicle, VV116, nirmatrelvir (with 50 mpk ritonavir), or the VV116-nirmatrelvir combination (Fig. 4a). The results showed that the lung viral loads of the VV116 (50 mpk and 100 mpk) groups and the nirmatrelvir (100 mpk) group were reduced by 1–2 log compared to the vehicle group at both 2 dpi and 4 dpi (Fig. 4b, c). The co-administration of VV116 (50 mpk) and nirmatrelvir (100 mpk, with 50 mpk ritonavir) (Combo) significantly reduced the viral loads in the lungs more dramatically than either VV116 or nirmatrelvir alone at both 2 dpi and 4 dpi, reducing the viral loads by 3–4 log-fold compared to that of the vehicle group (Fig. 4b, c). The detection of the viral titers in the lungs revealed that 50 mpk VV116 had a higher potency than 100 mpk nirmatrelvir at 4 dpi, and the 100 mpk VV116 and Combo treatments suppressed the viral titers in the lungs to below the limit of detection (Fig. 4d, e). Viral infection dramatically increased the expression of IL-6 and CCL2 in the lungs, while VV116, nirmatrelvir, and combination treatments resulted in a reduction in these cytokines during the initial days (Fig. 4f). At day 4, the expression of the cytokines remained high in the vehicle group, in contrast to that of the VV116, nirmatrelvir, and combination treatments (Fig. 4g). In brain tissues, drug or drug combination treatments significantly attenuated the expression of IL-1β, IL-6, TNF-α, CCL2, and ISG15 compared to that of vehicle group at day 4 (supplementary Fig. 5). Pathological images and immunofluorescence staining for detecting SARS-CoV-2 nucleocapsid protein also showed inflammation attenuation and viral load reduction in the lungs of the treatment groups compared to those of the vehicle group (Fig. 4h–l).

### Pharmacokinetics of the drug combination following oral administration

Next, a pharmacokinetic study was performed for VV116, nirmatrelvir (with ritonavir), and the VV116–nirmatrelvir combination in C57BL/6J mice with a dosage regimen consistent with that of the in vivo antiviral study (Fig. 4m, n). Following oral administration, VV116 was exclusively metabolized to the parent nucleoside X1, which reached its *C*_max_ with a value of 10,700 ± 100 ng/mL (corresponding to 36.6 ± 0.34 µM) at 0.25 h and had an AUC0-t of 19,484 ± 1109 ng h/mL. The plasma exposure was slightly lower than that in Balb/c mice orally receiving VV116 at a dose of 50 mg/kg in our previous study.^20^ Generally, nirmatrelvir (100 mg/kg) combined with ritonavir (50 mg/kg) showed a similar PK profile to that of VV116 in terms of *T*_max_ and *T*_1/2_. However, the *C*_max_ (45,833 ± 1537 g/mL, corresponding to 91.8 ± 3.1 µM) and the AUC0-t (174,969 ± 19,881 ng h/mL) of nirmatrelvir were much higher than those of X1. Regarding the VV116-nirmatrelvir combination, the plasma exposures of X1 and nirmatrelvir showed decreases (of 13.5% and 19.8%, respectively) compared with that in the individual study, as did the plasma *C*_max_ (of 20.3% and 12.5%, respectively).

## Discussion

The continuing COVID-19 pandemic has caused nearly 7 million deaths in the world, and it more likely posts severe threats to immunocompromised patients and elderly who have chronic diseases. Another concern of SARS-CoV-2 is that the emergency of more immune-evasive variants such as XBB lineage evade all human monoclonal antibodies in clinical therapy.^21,22^ In the past 2 years, a wave of drug discovery efforts has been performed to discover potent antivirals to combat SARS-CoV-2 and its variants, and most authorized drugs such as remdesivir, Paxlovid, molnupiravir, and VV116 exert broad-spectrum antiviral effects by targeting viral RdRp or 3CLpro,^9,23–25^ which non-structural proteins (NSPs) are highly conserved among coronaviruses. One of the advantages of these small-molecular compounds is the ability to become weapons to fight future viral pandemics. In this study, we explored the antiviral spectrum of VV116 demonstrating VV116 is an ideal oral drug that can broadly suppress human coronavirus including SARS-CoV-2 and its variants, and potentially block re-emerging animal-to-human transmission coronaviruses. Drug combination screening revealed that VV116 plus nirmatrelvir combination is an enhanced option in coronavirus treatment benefiting not only synergistic activity but also a high resistant barrier.

As a tri-isobutyrate ester prodrug, VV116 metabolizes into its parent nucleoside X1 in cells, and X1 showed anti-CoV activity comparable to that of GS-441524 (Fig. 1). X1 is a deuterated form of GS-4415254, which molecular has been proved that its triphosphate metabolite can efficaciously act by inducing the delayed chain termination of the nascent viral RNA chain targeting RdRp.^26^ Deuteration of GS-441524 may confer potential pharmacokinetic benefits.^9^ Due to the positive outcomes resulting from clinic trials,^10,27^ VV116 has been authorized in Uzbekistan and China for COVID-19 treatment. The current results further presented that VV116 broadly inhibited human coronaviruses including SARS-CoV-2 VOCs. In K18-hACE2 transgenic mice and Balb/c suckling mice, we validated the in vivo potency of VV116 against the SARS-CoV-2 Delta variant and HCoV-OC43 in a dose-dependent manner, respectively.

Furthermore, we applied a quantifying model to screen 20 double-target drug combinations and found that the VV116 and nirmatrelvir combination showed synergistic antiviral activity at the indicated concentrations. Both RdRp and 3CLpro play critical roles in the coronavirus life cycle, and drug combinations that inhibit both of them as distinct targets are expected to produce synergistic effects in most anticancer and antipathogen therapy cases.^28^ However, drug combinations against SARS-CoV-2 Delta targeting RdRp and 3CLpro reportedly presented additive antiviral effects rather than synergistic effects.^29^ Consistently, in our results, most drug combinations against RdRp-proteinase targets did not show higher anti-OC43 activity in RD cells at the indicated concentrations than their respective theoretic Bliss-dependent activities. Interestingly, among these combinations, VV116 presented a synergistic effect with nirmatrelvir at 2 × IC_50_ concentrations; by contrast, the remdesivir and nirmatrelvir combination did not display synergy at the relevant concentrations. The mechanism by which VV116 but not remdesivir synergizes with nirmatrelvir against HCoV-OC43 in RD cells is not yet known. A combination analysis in the zero-interaction potency (ZIP) model confirmed the synergistic antiviral activity of the VV116 and nirmatrelvir combination against HCoV-OC43 and the SARS-CoV-2 Delta variant in RD and Vero E6 cells, respectively.

Importantly, we found that oral co-administration of VV116, nirmatrelvir, and ritonavir effectively suppressed HCoV-OC43 replication in suckling mice, and the combination showed significantly higher antiviral potency than that of VV116 or nirmatrelvir alone (with ritonavir) at equal doses, especially in the lungs and spinal cord. The combination also presented synergistic potency in suppressing SARS-CoV-2 Delta variant replication in the lungs of K18-hACE2 mice. A previous in vivo study on K18-hACE2 mice infected with SARS-CoV-2 showed that co-administering molnupiravir and nirmatrelvir reduced the viral titer (TCID50) in the lungs by 1.4 log to 10^5^ TCID50.^30^ In contrast, in this study, the VV116–nirmatrelvir–ritonavir combination reduced the viral titer from 10^6^ PFU/g to an undetectable titer level. However, this result cannot prove that molnupiravir is inferior to VV116 in combination with nirmatrelvir for inhibiting SARS-CoV-2 at the different doses used in the two studies. Future head-to-head in vivo comparisons in vivo need to be performed. Co-administration of VV116, nirmatrelvir, and ritonavir in C57BL/6J mice did not significantly change the VV116 or nirmatrelvir exposure when they were administered alone, suggesting that the combination is potentially safe in terms of clinical pharmacokinetics. Considering in vitro selection studies showed that SARS-CoV-2 can acquire mutations that confer resistance to remdesivir and nirmatrelvir,^31–34^ VV116 plus nirmatrelvir combination therapy targeting both viral RdRp and 3CLpro not only enhances the antiviral efficacy but also potentially adds the resistant barrier of monotherapy.

In summary, we conducted an investigation and discovered that VV116 had broad-spectrum antiviral potency against SARS-CoV-2 variants of concern (VOCs), HCoV-OC43, and HCoV-229E in various cell lines. In vitro drug combination screening targeted RdRp and 3CLpro, highlighting the synergistic effect of VV116 and nirmatrelvir on HCoV-OC43 and SARS-CoV-2. When coadministered with ritonavir, the combination of VV116 and nirmatrelvir showed significantly enhanced antiviral potency with noninteracting pharmacokinetic properties in mice. Our findings will facilitate clinical treatment with VV116 or a VV116 + nirmatrelvir combination to fight coronavirus infection.

## Materials and methods

### Cell lines and viruses

African green monkey kidney Vero E6 cells (ATCC-1586), RD, Huh-7, HEK293T-hACE2-TMPRSS2 were maintained in Dulbecco’s modified Eagle’s medium (DMEM) with 10% fetal bovine serum (FBS) and 1% penicillin–streptomycin antibiotics. MRC5 was maintained in Minimum Essential Medium (MEM) with 10% fetal bovine serum (FBS) and 1% penicillin–streptomycin antibiotics. Cells were kept at 37 °C in a 5% CO_2_ atmosphere. The strains Delta variant (B.1.617.2, IVCAS6.7585), omicron BA.1 variant (IVCAS6.7600), and omicron BA.5 variant (IVCAS6.8981) of SARS-CoV-2 were obtained from National Virus Resource Center, and the strains HCoV-OC43 (ATCC VR-1588) and HCoV-229E (ATCC VR-740) were gifted from Wuhan University. The SARS-CoV-2 strains, HCoV-OC43, and HCoV-229E were propagated in Vero E6, RD, and MRC5 cells, respectively. All experiments with authentic SARS-CoV-2 viruses were carried out in the Biosafety Level 3 facility (BSL-3) of the Wuhan Institute of Virology, Chinese Academy of Sciences (CAS).

### Determination of antiviral activity in vitro

For SARS-CoV-2 variants, Vero E6 or HEK293T-hACE2-TMPRSS2 cells were pre-seeded to 48-well plates (50,000 cells/well). RD, Huh-7, or MRC5 cells (50,000 cells/well) were pre-seeded to 48-well plates for HCoV-OC43 and HCoV-229E infection. Cells were pre-seeded overnight, then the culture medium was removed and replaced with a medium containing a gradient concentration of compounds for 1 h incubation. Thereafter, cells were then inoculated with the Delta variant, omicron BA.1 variant, and omicron BA.5 variant of SARS-CoV-2 in a BSL-3 facility at a multiplicity of infection (MOI) of 0.01. HCoV-OC43 and HCoV-229E were inoculated in cells at MOI of 0.1 and 0.5, respectively. At 24 h (SARS-CoV-2 variants) or 48 h (HCoV-OC43 and HCoV-229E) after infection, the supernatant was collected for viral RNA copy number determination using real-time fluorescence quantitative PCR (qRT-PCR). The primers of qRT-PCR of SARS-CoV-2 Delta variant were RBD-qF1: 5’-CAATGGTTTAACAGGCACAGG-3’ and RBD-qR1: 5’-CTCAAGTGTCTGTGGATCACG-3’, of SARS-CoV-2 omicron BA.1 and BA.5 were RBD-Omi-qF1: 5’-CAATGGTTTAAAAGGCACAGG-3’ and RBD-qR1: 5’-CTCAAGTGTCTGTGGATCACG-3’, of HCoV-OC43 were OC43-NP-F: 5’-CGATGAGGCTATTCCGACTAGGT-3’ and OC43-NP-R: 5’-CCTTCCTGAGCCTTCAATATAGTAACC-3’,^35^ of HCoV-229E were 229E-NP-F: 5’- CAGTCAAATGGGCTGATGCA-3’ and 229E-NP-R: 5’- AAAGGGCTATAAAGAGAATAAGGTATTCT-3’. The inhibition rate of compounds was calculated based on the viral copy number, and the 50% effective concentration (EC_50_) was calculated with Graphpad Prism software 8.0. These experiments were independently performed three to six times.

### Quantifying antiviral activity of combinations of double drugs for screening high potency drug combinations against coronavirus

In this study, we applied a quantitative model which exerted in antivirals studies of HIV and HCV for evaluating the inhibitory activity of individual drugs and double-drug combinations against HCoV-OC43.^15,36^ Firstly, we treated HCoV-OC43 in RD cells with individual drugs of two classes: the nucleotide analogs including VV116, remdesivir, ribavirin, and NHC, the proteinase inhibitors including nirmatrelvir, boceprevir, paritaprevir, simeprevir, and lopinavir. RD cells (50,000 cells/well) were pre-seeded to 48-well plates overnight, followed by the culture medium was removed and replaced with a medium containing a gradient concentration of the drugs for 1 h incubation. Then HCoV-OC43 was inoculated in cells at MOI of 0.1 and the supernatant was collected for viral RNA copy number determination using real-time fluorescence quantitative PCR (qRT-PCR) using the primers described above at 48 hpi. The fractions of virus unaffected by the drugs (*f*_u_) were calculated with the formula: *f*_u_ = (viral copies in drug-treated supernatant)/(viral copies in mock-treated supernatant). Afterward, the median effect plots were produced by the relation between log [drug concentrations (*D*)] and log [(1−*f*_u_)/*f*_u_], in which the drug concentration that inhibited 50% viral replication (IC_50_) and the slope parameter (*m*) reflecting the steepness of the dose–response curve were gained (Supplementary Fig. 2 and Supplementary Table 2). The antiviral activity of an individual drug was indicated as the instantaneous inhibitory potential (IIP) with the following formula:

$${\rm{IIP}}={\rm{log }}(1/{{f}}_{{\rm{u}}})={\rm{log }}[1+{({D}/{{\rm{IC}}}_{50})}^{{\rm{m}}}]$$

Further, the antiviral activity of 20 double-drug combinations was evaluated in the same model. Here, drugs were combined from their initial concentrations (*D*_0_ = 0.25 × IC_50_) to their concentrations both increasing up to 8 × IC_50_. The IIP values of double-drug combinations (IIP_com_) were calculated from the formula: IIP_com_ = log (1/*f*_u-com_), where *f*_u-com_ is the experimental measurement of a drug combination. For evaluating whether drug combination exhibited synergistic effect or not, Bliss independence of each drug combination was predicted for comparison. Bliss independence assumes that each drug acts on different targets, what the principle corresponds to our double-drug combination designation, and is defined as:

$${{f}}_{{\rm{u}}-{\rm{com}}}({\rm{Bliss}})={{f}}_{{\rm{u}}}({A})\times {{f}}_{{\rm{u}}}({B})$$

*f*_u-com_ (Bliss) is the theoretic fraction of viral copies unaffected by the drug combinations (A + B), and *f*_u_ (A) and *f*_u_ (B) are the experimental fractions of viral copies unaffected by individual drug A and individual drug B, respectively. The Bliss independence IIP of drug combinations (IIP_com_) was calculated according to the formula described above.

### In vitro synergy analysis of the combination of VV116 and nirmatrelvir

RD cells were pre-seeded into 48-well plates (50,000 cells/well) and then incubated with DMEM with 10% fetal bovine serum (FBS) and 1% penicillin–streptomycin antibiotics overnight. Thereafter, cells were incubated with replaced medium containing gradient concentration of the two compounds, VV116 and nirmatrelvir, separately or in combination for 1 h before being infected with HCoV-OC43 at an MOI of 0.1 or SARS-CoV-2 Delta variant at an MOI of 0.01. At 24 h (SARS-CoV-2 Delta variants) or 48 h (HCoV-OC43) after infection, the supernatant was collected for viral RNA copy number determination using real-time fluorescence quantitative PCR (qRT-PCR) using the primers described above. The percentage of infection was normalized to that of the DMSO-treated control as 100% infection. The synergy δ-score was calculated using SynergyFinder, an R programming application, using the zero interaction potency (ZIP) model.^37^ The compounds were likely to be synergetic at a certain range when the δ-score was larger than 10. Viral infections were performed in a biosafety level 3 (BSL-3) facility for SARS-CoV-2 Delta variants or a biosafety level 2 (BSL-2) facility for HCoV-OC43. These experiments were independently performed three times.

### In vivo efficacy against HCoV-OC43 in suckling mice

The BALB/c mice were bred and maintained in a specific-pathogen-free (SPF) environment at the Laboratory Animal Center of Wuhan Institute of Virology, CAS. The pregnant mice with the same expected delivery date were acclimated in individually ventilated cages in an SPF environment under standard conditions. Food and water were available ad libitum. On the 4th day after birth, suckling mice were divided randomly into 10 groups, the vehicle group, the health group, the group receiving EIDD-2801 200 mpk orally quaque die (QD), the group receiving VV116 10 mpk orally QD, the group receiving VV116 25 mpk orally QD, the group receiving VV116 50 mpk orally QD, the group receiving nirmatrelvir 10 mpk with ritonavir 50 mpk orally QD, the group receiving nirmatrelvir 25 mpk with ritonavir 50 mpk orally QD, the group receiving drug combination of VV116 10 mpk and nirmatrelvir 10 mpk with ritonavir 50 mpk orally QD, the group receiving drug combination of VV116 25 mpk and nirmatrelvir 25 mpk with ritonavir 50 mpk orally QD. Mice were anesthetized by isoflurane inhalation and then intranasally infected with 1 × 10^4^ TCID50 of HCoV-OC43. Two hours after viral infection, mice were orally treated with vehicle, individual drugs, or drug combination according to the group description as described above (day 0). Mice were orally treated in the following days as described above. Body weight changes were measured daily. On Day 5, every mouse in each group was sacrificed for virological and histopathological analyses. Multiple organs and tissues from mice, including brains, spinal cords, lungs, and kidneys, were collected on ice. Part of the organs and tissues were homogenized with DMEM and subsequently centrifuged at 3000 rpm for 10 min at 4 °C. Viral and host RNA from the organs and tissues were extracted with the RNeasy Mini Kit (Qiagen) and reverse transcribed (PrimeScript Reverse Transcriptase, Takara) according to the operation instruction, then the absolute viral RNA copy and relative host cytokine mRNA expression in the organs and tissues was detected quantitatively by real-time fluorescence quantitative PCR. The viral RNA copies were calculated by the concentration of standard plasmids for the HCoV-OC43 membrane proteins. The primers of qRT-PCR for viral RNA copies were OC43-M-F: 5’-GGCTTATGTGGCCCCTTACT-3’ and OC43-M-R 5’-GGCAAATCTGCCCAAGAATA-3’. The primers of qRT-PCR for host cytokine mRNA expression were IL1B-qF1: 5’-TTGACGGACCCCAAAAGATG-3’, IL1B-qR1: 5’-AGAAGGTGCTCATGTCCTCA-3’; IL6-qF1: 5’-GTTCTCTGGGAAATCGTGGA-3’, IL6-qR1: 5’-TGTACTCCAGGTAGCTATGG-3’; IFNAR1-qF1: 5’-TCGTGGAATGAGGTTGATCCG-3’, IFNAR1-qR1: 5’-CCCACATGTTCCCGTCTTGT-3’; TNF-α-qF1: 5’-ATCGGTCCCCAAAGGGATGA-3’, TNF-α-qR1: 5’-GCTCCTCCACTTGGTGGTTT-3’; CCL2-qF1: 5’-CACCAGCCAACTCTCACTGAA-3’, CCL2-qR1: 5’-GTGGGGCGTTAACTGCATCT-3’; CXCL10-qF1: 5’-GGTCTGAGTGGGACTCAAGG-3’, CXCL10-qR1: 5’-GTGGCAATGATCTCAACACG-3’; ISG15-qF1: 5’-GGTGTCCGTGACTAACTCCAT-3’, ISG15-qR1: 5’-TGGAAAGGGTAAGACCGTCCT-3’; GAPDH-qF1: 5’-TGGTGAAGGTCGGTGTGAAC-3’, GAPDH-qR1: 5’-GAAGGGGTCGTTGATGGCAA-3’. For histologic examination, mouse organs and tissues were collected directly after euthanasia and placed in 4% paraformaldehyde for >5 days after which tissues were embedded in 3.5-mm paraffin. Fixed tissue samples were used for hematoxylin–eosin (H&E) and immunofluorescence staining for the detection of the HCoV-OC43 antigen (anti-HCoV-OC43 nucleocapsid protein rabbit serum, ABclonal). The image information was collected using a Pannoramic MIDI system (3DHISTECH, Budapest) and FV1200 confocal microscopy (Olympus). The animal experiments conformed to the use and care of laboratory animals and were approved by the ethics committee of Wuhan Institute, CAS. Viral infections were performed in an animal biosafety level 2 (BSL-2) facility.

### In vivo efficacy against SARS-CoV-2 Delta variant in K18-hACE2 mice

Age of 7–8 weeks K18-hACE2 male mice were purchased from Jiangsu GemPharmatech Biotechnology Co., Ltd. (Jiangsu, China). The animal experiments conformed to the use and care of laboratory animals and were approved by the ethics committee of Wuhan Institute, CAS. Viral infections were performed in biosafety level 3 (BSL-3) facility. Animals were divided into five groups (*n* = 9 for each group), the vehicle group, the group receiving VV116 100 mpk, the group receiving VV116 50 mpk, the group receiving nirmatrelvir 100 mpk with ritonavir 50 mpk, the group receiving drug combination (Combo) of VV116 50 mpk and nirmatrelvir 100 mpk with ritonavir 50 mpk. Mice were anesthetized by isoflurane inhalation and then intranasally infected with 50 µl 1 × 10^3^ PFU/ml of SARS-CoV-2 Delta variant. Two hours after viral infection, mice were orally treated with vehicle, individual drugs, or drug combination according to group description as described above (day 0). Mice were treated twice at 8 h intervals daily in the following days. On day 2, part of each group (5 mice) was sacrificed, and the lung tissues were collected for viral copies detection and immunofluorescence stain analysis. On day 4, the left mice of each group were sacrificed and performed following the same procedure. Viral RNA from the lung tissues was extracted with the RNeasy Mini Kit (Qiagen) and reverse transcribed (PrimeScript Reverse Transcriptase, Takara) according to the operation instruction, then the absolute viral RNA copy in the tissue was detected quantitatively by real-time fluorescence quantitative PCR. The viral RNA copy was calculated by standard plasmid concentration. For histologic examination, mouse lungs and brains were collected directly after euthanasia and placed in 4% paraformaldehyde for >5 days after which tissues were embedded in 3.5-mm paraffin. Fixed tissue samples were used for hematoxylin–eosin (H&E) and immunofluorescence staining for the detection of the SARS-CoV-2 antigen (SARS-CoV-2 Nucleocapsid Protein (HL344) Rabbit mAb #26369, CST). The image information was collected using a Pannoramic MIDI system (3DHISTECH, Budapest) and FV1200 confocal microscopy (Olympus).

### Immuno-plaque assay

RD cells were pre-seeded onto 24-well plates and incubated at 37 °C for 24 h until 90% confluence. The supernatants of homogenized organs and tissues were 10-fold serially diluted in DMED. Subsequently, 50 μl of each dilution was added to the wells. After incubating for 1 h at 37 °C, the inoculum was removed and 200 μl of the overlay medium (DMEM with 2% FBS and 1% carboxymethyl cellulose) was added to the wells. Four days post-infection, cells were fixed with 4% paraformaldehyde for 2 h followed by washing with phosphate-buffered saline (PBS) three times. Fixed cells were permeabilized with 0.5% Triton X-100 for 30 min and successively blocked with 5% bovine serum albumin (BSA) for 30 min at room temperature. The cells were probed using anti-HCoV-OC43 nucleocapsid protein rabbit serum (ABclonal) as the primary antibody and HRP-conjugated Anti-Rabbit IgG (SA00001-2, Proteintech) as the secondary antibody and then stained with a DAB staining kit (PA110, TIANGEN).

### Pharmacokinetic studies of VV116, nirmatrelvir + ritonavir and VV116 + nirmatrelvir + ritonavir in C57BL/6J mice

The PK studies were conducted at Suzhou HQ Bioscience Co., Ltd. Eighteen C57BL/6J mice (*N* = 6 for each group, male) were randomly divided into three groups, and fasted for 12 h before dosing. The three groups received oral dose of VV116, nirmatrelvir + ritonavir and VV116 + nirmatrelvir + ritonavir at 50 mg/kg, 100 + 50 mg/kg and 50 + 100 + 50 mg/kg, respectively. The vehicle for oral administration of the test compounds was 5% DMSO + 5% Solutol HS-15 + 5% PEG400 + 85% Saline. Blood sample (70 μL) was collected from the orbit of the first three mice in each group at 0.25, 1.0, 4.0, and 8.0 and the last three mice in each group at 0.5, 2.0, 6.0, and 24 h post-dosing. The sample was taken into EDTA-K2 tubes, and centrifuged at 11,000 rpm for 5 min. The plasma was separated and frozen in a refrigerator at −70 °C for testing. The operation was conducted under an ice water bath. The concentration of analyte in plasma was determined by LC–MS/MS.

### Supplementary information

Supplementary Information

## Data Availability

All data supporting the findings in this article are available in the main text and the Supplementary Materials.
