# Supplementary material for: A viral RNA-dependent RNA polymerase inhibitor VV116 broadly inhibits human coronaviruses and has synergistic potency with 3CLpro inhibitor nirmatrelvir
Source: Signal Transduct Target Ther. 2023 Sep 22;8:360. doi: 10.1038/s41392-023-01587-1 (PMC10514301; doi:10.1038/s41392-023-01587-1)
Supplement: Supplementary file 1 — Supplementary Information [file 41392_2023_1587_MOESM1_ESM.docx]

Supplementary Materials for

A viral RNA-dependent RNA polymerase inhibitor VV116 broadly inhibits human coronaviruses and has synergistic potency with 3CLpro inhibitor nirmatrelvir

Yumin Zhang, Yuan Sun, Yuanchao Xie, Weijuan Shang, Zhen Wang, Hualiang Jiang, Jingshan Shen, Gengfu Xiao, Leike Zhang

Correspondence to: Leike Zhang (zhangleike@wh.iov.cn), Gengfu Xiao (xiaogf@wh.iov.cn), Jingshan Shen (shenjingshan@simm.ac.cn)

**This PDF file includes:**

Supplementary Figs. 1 to 5

Supplementary Tables 1 to 2

supplementary Fig. 1

The cytotoxicity of VV116, N1 (X1), remdesivir (RDV), GS-441524, and β-d-N4-hydroxycytidine (NHC) in Vero E6, Huh-7, MRC-5, RD, and HEK293T-ACE2-TMPRSS2 cells.

supplementary Fig. 2

Median effect plots for the activity of nucleotide analogs (VV116, remdesivir, NHC, ribavirin) and viral protease inhibitors (nirmatrelvir, boceprevir, paritaprevir, simeprevir, lopinavir) against HCoV-OC43 in RD cells. Each point represents one of three independent assays carried out by real-time fluorescence quantitative PCR targeting HCoV-OC43 N protein gene which in the supernatants. D, drug concentration; fu, the fraction of virus unaffected by drug calculated with the formula: fu = (viral copies in drug-treated supernatant) / (viral copies in mock-treated supernatant).

supplementary Fig. 3

The interaction landscape of VV116 and nirmatrelvir against the SARS-CoV-2 Omicron BA.5 variant in Vero E6 cells. The synergy δ-score was calculated using SynergyFinder with the zero-interaction potency (ZIP) model.

**
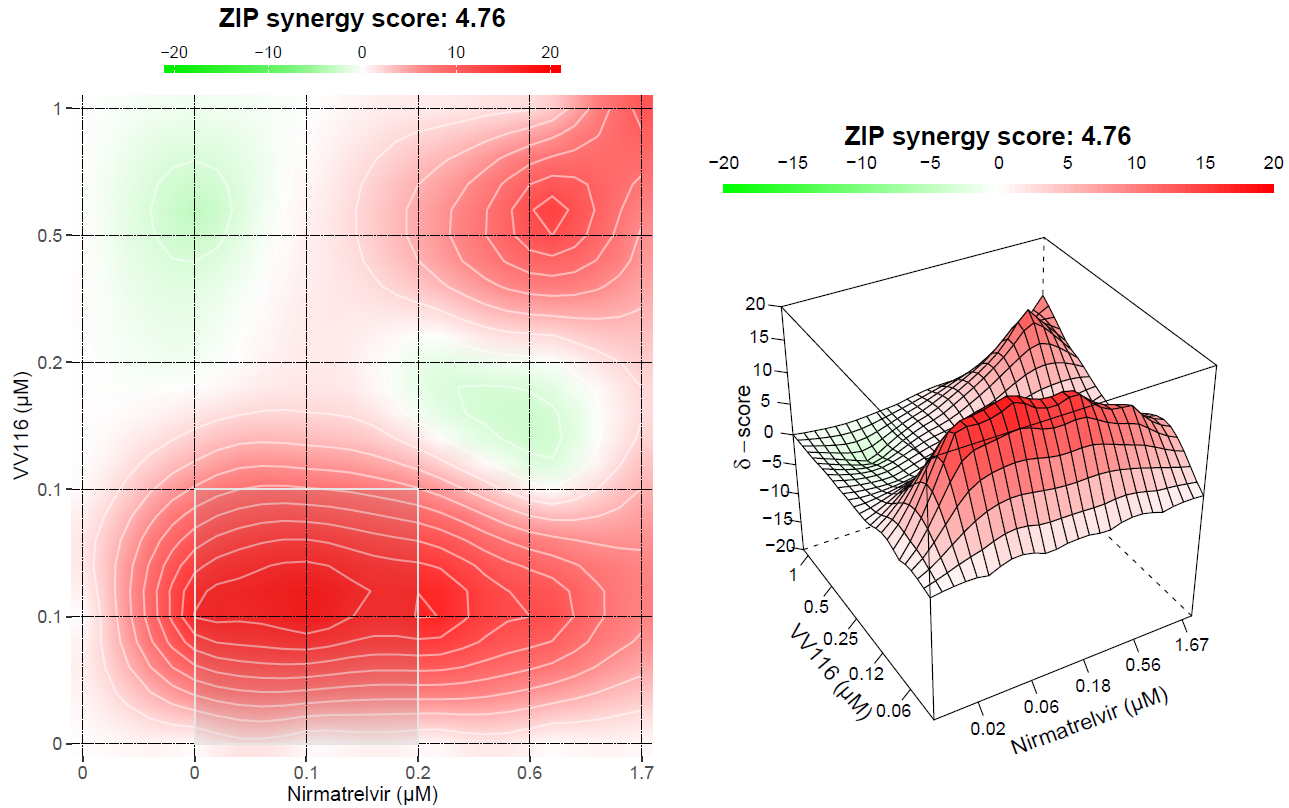
**

supplementary Fig. 4

Histopathology and tissue immuno-fluorescence assay results of HCoV-OC43 infected 5-day old suckling BALB/c mice treated with different does of VV116, nirmatrelvir or VV116 plus nirmatrelvir combination. **a-i**, the lung sections from HCoV-OC43 infected mice were H&E strained for histopathology analyses. In the immuno-fluorescence assay, the nuclei were strained with DAPI (blue) and the HCoV-OC43 nucleocapsid proteins were strained with Alexa Fluor 488 (green) to show the site of infection. **a**: vehicle; **b**: VV116-10mpk; **c**: VV116-25mpk; **d**: VV116-50mpk; **e**: nirmatrelvir-10mpk + rito-50mpk; **f**: nirmatrelvir-25mpk + rito-50mpk; **g**: Combo 1; **h**: Combo 2; **i**: EIDD2801-200mpk. Scale bars on the pictures of tissue slides indicate 400 µM.

supplementary Fig. 5

Cytokine gene expression was measured in brains infected with SARS-CoV-2 delta variant at day 2 and day 4. The relative gene expression of IL-1β, IL-6, IFNAR, TNF-α, CCL2, CXCL10, and ISG15 was compared to that of unchallenged mice.

supplementary Table 1

The selectivity index (SI) of VV116, X1, remdesivir (RDV), GS-441524, and β-d-N4-hydroxycytidine (NHC) against SARS-CoV-2 (delta, omicron BA.1, omicron BA.5), HCoV-OC43, and HCoV-229E in Vero E6, HEK293T-ACE2-TMPRSS2, RD, Huh-7, and MRC5 cells.


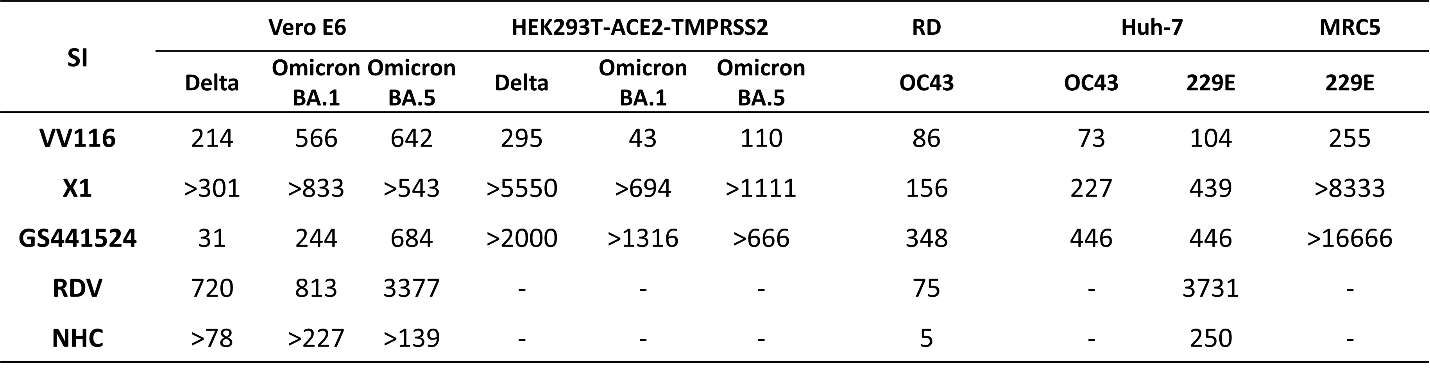


supplementary Table 2

The parameters of the slope steepness (m) and the drug concentration that inhibit 50% viral replication (IC_50_) of the selected drugs.

|  | **m** | **IC_50_ (µM)** |
| --- | --- | --- |
| **VV116** | 5.53 | 2.16 |
| **RDV** | 2.40 | 0.0202 |
| **NHC** | 2.80 | 5.68 |
| **Ribavirin** | 2.19 | 49.5 |
| **Nirmatrelvir** | 4.49 | 0.121 |
| **Paritaprevir** | 1.55 | 2.50 |
| **Simeprevir** | 2.18 | 2.22 |
| **Lopinavir** | 1.94 | 1.44 |
| **Boceprevir** | 11.2 | 26.9 |
